# Use of Graphite Oxide and Graphene Oxide as Catalysts in the Synthesis of Dipyrromethane and Calix[4]pyrrole

**DOI:** 10.3390/molecules16097256

**Published:** 2011-08-25

**Authors:** Shive Murat Singh Chauhan, Sweta Mishra

**Affiliations:** Bioorganic Research Laboratory, Department of Chemistry, University of Delhi, Delhi-110007, India; E-Mail: sweta.chem19@gmail.com

**Keywords:** graphite oxide, graphene oxide, 5,5-dimethyldipyrromethane, calix[4]pyrrole, solid acid

## Abstract

Graphite oxide and graphene oxides have been used as solid catalysts for the synthesis of 5,5-dialkyldipyrromethanes and calix[4]pyrroles in organic and aqueous solutions at room temperature.

## 1. Introduction

Graphene is a two dimensional sheet of sp^2^ hybridized carbon with remarkable thermal, mechanical and electronic properties [1,2,3,4]. Top down is an important method for the preparation of graphene by oxidation of graphite [5], as compared to other methods [6,7]. Graphite oxides containing OH, epoxy and carboxyl groups have been prepared by oxidation of graphite via minor modification of known methods [8,9] and their structures have been characterized by different spectroscopic methods [10,11,12]. The presence of polar groups is responsible for the acidic solutions formed when graphite and graphene oxides are suspended in aqueous media [13,14]. In organic synthesis polycarbon acids [15,16] and polyacids on carbon nanostructures [17,18,19,20] are considered more robust in aqueous as well as organic solvents than other solid acids [20,21,22,23] such as ion exchange resins [24], heteropolyacids [25,26,27] and layer transition metal oxides [28,29]. 

One of the most important methods developed for the mass production of graphene is exfoliation of graphite oxide in aqueous solution to single layers of graphene oxide. This exfoliation can be performed in aqueous solution alone or in presence of surfactants [30], polymers [31,32], ionic liquids [33] and polar solvents [34,35]. Aqueous suspensions of graphene oxide have been used in the oxidation of alcohols and *cis*-stilbene and the hydration of various alkynes to the corresponding aldehydes, acids and ketones under mild conditions [36,37]. The electrical conductivity of the graphene oxide differs from that of pristine graphene, hence various attempts have been made to deoxygenate graphene oxide to the maximum extent to regain the aromaticity and electrical conductivity. The hydroxyl and the epoxy groups and some carboxylic groups can be removed by reduction of graphite oxide by hydrazine, ascorbic acid and other reducing agents [38,39,40]. 

**Scheme 1:**
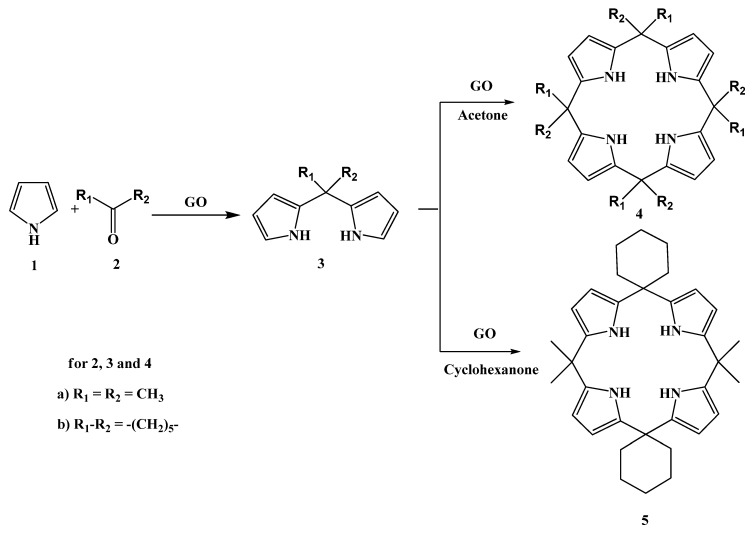
Synthesis of dipyrromethane and calix[4]pyrrole.

Porphyrinogens and more stable calix[4]pyrroles are important tetrapyrrolic macrocycles used in biosynthesis of porphyrinoids [41], supramolecular chemistry [42], anion receptor [43,44] and material chemistry [45,46,47]. They are synthesized by condensation of pyrroles with dialkylketones in the presence of aqueous acids [48,49], Lewis acids [50,51] and solid acids including zeolites [52], molecular sieves [53,54] and Amberlyst-15 [55] under different reaction conditions. Although various solid acids have been used in the synthesis of different calix[4]pyrroles, to the best of our knowledge graphite oxide and graphene oxides have not been used for this purpose. Hence, we report the synthesis of selected calix[4]pyrroles in aqueous suspensions of graphite oxide and graphene oxides under different reaction conditions (Scheme 1). 

## 2. Results and Discussion

The reaction of acetone (**2a**) with pyrrole (**1**) in a suspension of graphite oxide (GO) in dichloromethane at room temperature gave dipyrromethane (**3a**) as the major product. The structure of **3a** was confirmed by spectroscopic data, mixed melting point [56] and comparison of HPLC retention times with an authentic sample (Table 1). The reaction of **1** with cyclohexanone (**2b**) gave the corresponding dipyrromethane **3b** in 70% yield. An analogous condensation of pyrrole and **2b** in dichloromethane in the presence of the solid acid zeolite HY gave dipyrromethane **3b** in 63% yield, along with other products, whereas the same reaction in the presence of HZSM in dichloromethane gave dipyrromethane **3b** and calix[4]pyrrole **4b** in 53 and 11% yield, respectively [52]. The reaction of **1** with **2a** in the presence of Al-MCM-41 zeolite gave **3a** and **4a** in 12 and 70% yield, respectively [52]. The reaction of the above in the presence of Amberlyst^TM^-15 gave **4a** and N-confused calix[4]pyrrole in 83 and 14% yield, respectively [55]. The high yield of **4a** in acetone may be explained by initial formation of **3a** which on subsequent reaction with acetone leads to calix[4]pyrrole. This was also confirmed by reaction of **3a** with **2b** with the formation of **5** in 17% yield. (Table 1) [52]. The reaction of **1** and **2a** was performed in other polar solvents and the results are reported in Table 1. A moderate yield of calix[4]pyrrole **4** was obtained when the reaction was performed in methanol or acetonitrile (Table 1). The graphite oxide is stable in organic solvents and it may be used several times without much loss of catalytic activity (Table 1, entry 14).

The formation of calix[4]pyrrole **4a** in excess acetone may be explained by initial formation of **3a** which on subsequent reaction with acetone leads to **4a**. The reaction of **3a** with cyclohexanone lead to cailx[4]pyrrole **5** (Scheme 1).

The reaction of **1** and **2a** in the presence of graphite oxide suspended in aqueous solution gave **3a** as major product in 92% yield (Table 2). The reaction of pyrrole and ketones regardless of the ratio of starting compounds, forms the dialkyldipyrromethanes in the presence of weak acid in aqueous solutions [48]. The increase in the ratio of acetone decreases the yield of **3a** and a minor amount of **4a** was observed (Table 2). 

The reaction of **1** and **2a** in the presence of graphite oxide and SDS gave dipyrromethane (48%), calix[4]pyrrole (35%) and N-confused calix[4]pyrrole in 10% yield at room temperature in 1.5 to 3 h (Table 3). 

The addition of SDS to graphite oxide in aqueous solution forms the corresponding acid which catalyzes the formation of dipyrromethane in low and calix[4]pyrrole in high yields. The graphite oxides have been dispersed in SDS and poly(sodium 4-styrenesulphonate) in aqueous solution [32]. The reactions of **1** with **2a** in the presence other surfactants and salts are given in Table 3. The reaction of **1** and **2a** in the presence of reduced graphene oxide [57,58] in aqueous solution gave **3a** as major product and **4a** as minor product (Table 4). 

The reduced graphene oxide agglomerizes in aqueous solution, but in the presence of organic solvents [59] and ionic liquids [60] it may be dispersed in aqueous solution. Suitable chemical modifications of reduced graphene oxides have been used to solubilize the graphene oxides in aqueous [60] and organic solvents [61]. The above results indicate that graphite and graphene oxide may be used as solid acids in various organic transformations.

## 3. Experimental

### 3.1. General

The infrared spectra (IR) were recorded on a Perkin-Elmer FT-1710 spectrophotometer. ^1^H-NMR spectra were recorded in CDCl_3_ on a Bruker Avance 400 MHz spectrophotometer with TMS as internal standard. UV-Vis spectra were recorded on Perkin-Elmer Lambda 35 spectrophotometer. Raman spectra were recorded on inVia Renishaw Raman spectrophotometer using a green (514 nm) laser. Powder XRD were recorded on a Bruker Discover 8 X-ray diffractometer. HPLC analysis was performed on a Waters 2998 using a Waters PAH C_18_ HPLC column (4.6 × 250 mm) and methanol as the eluent. Starting materials such as pyrrole (**1**) and acetone (**2**) were obtained from Acros USA and distilled immediately prior to use. The experimental operations were performed under ambient conditions. Neutral alumina was used for all the chromatographic purifications. Graphite powder was obtained from Alfa Aesar, USA. 

### 3.2. Preparation of Authentic Samples

*5,5-Dimethyldipyrromethane* (**3a**) was prepared by following the literature procedure starting from acetone and pyrrole in ionic liquid [56] and aqueous solution [48]; m.p. 56 °C (lit [56] 55–57 °C);^1^H- NMR: 1.61 (s, 6H, –CH_3_), 6.08 (s, 2H, β-pyrrole), 6.1 (d, 2H, β-pyrrole), 6.5 (d, 2H, α-pyrrole), 7.64 (s, br, 2H, NH-pyrrole); HPLC retention time = 3.1 min.

*Octamethylcalix[4]pyrrole* (**4a**) was prepared by following a literature procedure [55] starting from acetone and pyrrole; m.p. 294 °C (lit [55] 296 °C ); ^1^H-NMR: 7.01 (4H, br s, NH), 5.89 (8H, d, *J* = 2.5 Hz, β-pyrrole), 1.50 (24 H, s); HPLC retention time = 3.8 min.

### 3.3. Preparation of Catalysts

#### 3.3.1. Graphite Oxide

KMnO_4_ (9 g) was added in portions to a cooled (0 °C) solution of conc. H_2_SO_4_ (69 mL) containing graphite (3 g) and NaNO_3_ (1.5 g). The mixture was stirred at room temperature for 5 days. Distilled water (138 mL) was added slowly to the reaction mixture while the temperature was kept well below 98 °C for 3 h. The resultant bright-yellow suspension was diluted and a solution of H_2_O_2_ (6 mL, 30%) was added dropwise. The reaction mixture was centrifuged and washed to remove the remaining salts. The wet graphite oxide was dewatered by vacuum drying (50 °C). UV-Vis (λ_max_) (H_2_O) = 230 nm, (DMF) = 269 nm. FTIR (cm^−1^) = 3447 (OH), 1740 (C=O), 1636 (OH bending), 1091 (C-O). Raman spectra: 1350 (D band), 1584 (G band), D/G ratio: 0.85. XRD data: 10.5°.

#### 3.3.2. Graphene Oxide

Aqueous colloids of single layer graphene oxide nanosheets were produced by exfoliation of graphite oxide dispersed in deionized water with ultrasonication [32].

#### 3.3.3. Preparation of Reduced Graphene Oxide

Graphite oxide (75 mg) was dispersed in water (75 mL) with sonication. Sodium borohydride (600 mg) was added to the GO dispersion after the pH being adjusted to 9–10 with 5 wt% sodium carbonate solution. The mixture was then kept at 80 °C for 1 h under constant stirring. During reduction, the dispersion turned from dark brown to black accompanied by outgassing. UV-Vis (λ_max_) (H_2_O) = 270 nm. FTIR (cm^−1^) = 3440 (O-H), 1740 (C=O), 1091 (C-O). Raman spectra: 1350 (D band), 1584 (G band), D/G ratio: >1.

### 3.4. The Reaction of Pyrrole and Acetone in Organic Solvents

Equimolar amounts of pyrrole (14.4 mmol) and acetone (14.4 mmol) were taken up in dichloromethane (20 mL). Graphite oxide (10% w/w) was added in portions to the reaction mixture, which was stirred at ambient temperature for the appropriate time (as indicated in Table 1). The reaction progress was monitored by thin layer chromatography (TLC) with petroleum ether-chloroform. After the completion of reaction, the catalyst was removed by filtration and washed thoroughly with CH_2_Cl_2_ to dissolve all the contents. The filtrate was concentrated to give the crude product, which was subjected to column chromatography over neutral alumina eluting with petroleum ether-chloroform to afford pure calix[4]pyrrole and further elution of column gave dipyrromethane. The reaction mixture was analysed by HPLC. The HPLC yields are given in Table 1.

### 3.5. The Reaction of Pyrrole and Acetone in Aqueous Solution 

Equimolar amounts of pyrrole (14.4 mmol) and acetone (14.4 mmol) were taken up in water (20 mL). Graphite oxide (10% w/w) was added in portions to the above reaction mixture that was stirred at ambient temperature for the appropriate time (Table 2). The reaction progress was monitored by thin layer chromatography (TLC) with petroleum ether-chloroform. After completion of reaction, the catalyst was removed by extraction with CH_2_Cl_2_. The organic layer was separated dried over sodium sulfate, filtered and the filtrate was concentrated to give the crude product, which was subjected to column chromatography over neutral alumina eluting with petroleum ether-chloroform to afford pure calix[4]pyrrole and further elution of column gave dipyrromethane. The reaction mixture was analysed by HPLC. The HPLC yields are given in Table 2. 

### 3.6. The Reaction of Pyrrole and Acetone in Aqueous Solution in Presence of Suphonate Salts

Pyrrole (14.4 mmol), acetone (14.4 mmol) and surfactant/salt (0.034 mmol) were taken up in water (20 mL). Graphite oxide (10% w/w) was added in portions to the reaction mixture, which was stirred at ambient temperature for the appropriate time (Table 3). The reaction progress was monitored by thin layer chromatography (TLC) with petroleum ether-chloroform. After the completion of reaction, the catalyst was removed by extraction with CH_2_Cl_2_. The organic layer was separated dried over sodium sulfate, filtered and the filtrate was concentrated to give the crude product, which was subjected to column chromatography over neutral alumina eluting with petroleum ether-chloroform to afford pure calix[4]pyrrole and further elution of column gave dipyrromethane.The reaction mixture wasanalysed by HPLC. The HPLC yields are given in Table 3. 

### 3.7. The Reaction of Pyrrole and Acetone Catalyzed by Reduced Graphene Oxide under Different Conditions

Equimolar amounts of pyrrole (14.4 mmol) and acetone (14.4 mmol) were taken up in different solvents (20 mL). Graphene oxide (10% w/w) was added in portions to the above reaction mixture that was stirred at ambient temperature for the appropriate time (Table 4). The reaction progress was monitored by thin layer chromatography (TLC) with petroleum ether-chloroform. After completion of reaction, the catalyst was removed by filtration or extraction. Filtrate was concentrated to give the crude product, which was subjected to column chromatography over neutral alumina eluting with petroleum ether-chloroform to afford pure calix[4]pyrrole and further elution of column gave dipyrromethane.The reaction mixture was analysed by HPLC. The HPLC yields are given in Table 4. 

## 4. Conclusions

Graphite oxide and reduced graphene oxides have been used as solid acid catalysts for the room temperature preparation of dipyrromethanes and calix[4]pyrroles in organic and aqueous solutions.

## Figures and Tables

**Table 1:** Graphite oxide catalyzed condensation of pyrrole and acetone in different solvents ^a^.

Entry	Solvent	Catalyst	2	Conversion of pyrrole (%)	Yield of product (wt%) ^b,c^		
					3	4	Other
1	CH_2_Cl_2_	GO	**2a**	99	99	-	-
2	CH_2_Cl_2_^d^	GO	**2a**	99	99	-	-
3	CH_2_Cl_2_	GO	**2b**	90	73	2	15
4	CH_2_Cl_2_^d^	GO	**2b**	86	70	-	9
5	CH_2_Cl_2_^d^	Zeolite HY[52]	**2b**	87.9	62.7	-	16.2
6	CH_2_Cl_2_^d^	HZSM-5(30)[52]	**2b**	69.6	53.0	10.7	5.9
7	CH_2_Cl_2_^d^	Al-MCM-41	**2a**	95	12.3	70.3	12.4
8	CHCl_3_	Amberlyst^TM^-15[55]	**2a**	99		83	14 ^e^
9	CHCl_3_	GO	**2a**	99	97	2	-
10	CCl_4_	GO	**2a**	99	95	3	-
11	Methanol	GO	**2a**	100	90	10	-
12	CH_3_CN	GO	**2a**	98	80	13	5^e^
13	Ethylene glycol	GO	**2a**	98	34	7	57
14	Acetone ^g^	GO	**2a**	99	27	62 (50) ^f^	10^e^ (6) ^f^

^a^ *Reaction conditions*: pyrrole (14.4 mmol), acetone (14.4 mmol); graphite oxide (50 mg); solvent (20 mL); reaction time: 20h; ^b^ yields were calculated by HPLC; ^c^ other products may be tripyrromethane, open chain tetramer or N-confused calix[4]pyrrole; ^d^ reaction were performed in 10h in order to compare the results; ^e^ N-confused calix[4]pyrrole; ^f^ Isolated yield; ^g^ Catalyst was reused over three runs.

**Table 2:** Reaction of pyrrole and acetone in presence of graphite oxide in aqueous solution ^a^.

Entry	Pyrrole:Acetone	Conversion of pyrrole (%)	Yield of product (wt%) ^b,c^		
			3	4	Other
1	1:1	93	92	-	1
2	2:1	97	97	-	-
3	5:1	99	98	-	1
4	1:1.5	98	97	-	1
5	1:5	100	96	3	1
6	1:10	100	84	8	2 ^d^

^a^ *Reaction conditions*: water (20 mL); Graphite oxide (50 mg); reaction time: 20h; ^b^ yields were calculated by HPLC peak area; ^c^ other products may be tripyrromethane, open chain tetramer or N-confused calix[4]pyrrole; ^d^ N-confused calix[4]pyrrole.

**Table 3:** Reaction of pyrrole and acetone in the presence of graphite oxide and surfactant and salts in aqueous solution ^a^.

Entry	Surfactant and salts	Time	Conversion of pyrrole (%)	Yield of product (wt%) ^b,c^		
				3	4	Other
1	SDS alone	24 h	No reaction	-	-	-
2	SDS+GO	1.5–3 h	93	48	35	10 ^d^
3	CTAB + GO	24 h	96	93	-	3
4	PEG + GO	24 h	99	96	-	3
5	Poly(sodium 4-styrenesulfonate) + GO	24 h	89	84	5	-
6	Sodium methanesulfonate + GO	24 h	99	99	-	-
7	Methanesulfonic acid (2 × 10^−2^ M)	24 h	99	79	2	-
8	Sodium trifluoromethane sulfonate + GO	24 h	99	99	-	-

^a^ *Reaction conditions*: water (20 mL); graphite oxide (50 mg); surfactant and salts (50 mg); ^b^ yield were calculated by HPLC peak area; ^c^ other products may be tripyrromethane, open chain tetramer or N-confused calix[4]pyrrole; ^d^ N-confused calix[4]pyrrole.

**Table 4:** Reaction of pyrrole and acetone in presence of reduced graphene oxide ^a^.

Entry	Solvent	Time	Conversion of pyrrole (%)	Yield of product (wt%) ^b,c^		
				(3)	(4)	Other
1	Dichloromethane	24 h	99	98	1	-
2	Methanol	24 h	99	94	5	-
3	Acetone	24 h	100^c^	52	25	10 ^d^
4	Water	24 h	92	82	-	10
5	Water + SDS	24 h	92	69	1	22

^a^ *Reaction conditions*: solvent (20 mL); catalyst: reduced graphene oxide (50 mg); ^b^ yields were calculated by HPLC peak area; ^c^ other products may be tripyrromethane, open chain tetramer or N-confused calix[4]pyrrole; ^d^ N-confused calix[4]pyrrole.
